# Use of Intravenous Amiodarone in the Treatment of Nifekalant-Resistant Arrhythmia: A Review of 11 Consecutive Cases with Severe Heart Failure

**DOI:** 10.3390/ph4060794

**Published:** 2011-05-31

**Authors:** Koji Nakagawa, Kazufumi Nakamura, Kengo Fukushima Kusano, Satoshi Nagase, Takeshi Tada, Masato Murakami, Yoshiki Hata, Hiroshi Morita, Kunihisa Kohno, Kazumasa Hina, Tohru Ujihira, Tohru Ohe, Hiroshi Ito

**Affiliations:** 1 Department of Cardiovascular Medicine, Okayama University Graduate School of Medicine, Dentistry and Pharmaceutical Sciences, 2-5-1 Shikata-cho, Kita-ku, Okayama, 7008558, Japan; 2 Department of Cardiology, National Hospital Organization Iwakuni Medical Center, Iwakuni, 7400304, Japan; 3 Department of Cardiology, Okayama Heart Clinic, Okayama, 7008558, Japan; 4 Department of Cardiology, Okayama Redcross Hospital, Okayama, 7008558, Japan; 5 Department of Cardiology, Cardiovascular Center Sakakibara Hospital, Okayama, 7008558, Japan

**Keywords:** arrhythmias, heart failure, ventricular arrhythmia, atrial fibrillation

## Abstract

*Background*: Both nifekalant hydrochloride (NIF), a selective I_Kr_ blocker, and intravenous amiodarone (AMD), a multi-channel (including I_Kr_ blocking) blocker, have been reported to be efficacious for refractory arrhythmias. However, the optimal use of those antiarrhythmic drugs for refractory arrhythmia with severe heart failure has not been established. Intravenous AMD might be effective for arrhythmias refractory to NIF in patients with severe heart failure. Here, we report that intravenous amiodarone was effective in the treatment of nifekalant-resistant in a group of arrhythmia patients with severe heart failure. *Methods*: Eleven severe heart failure patients who had received intravenous AMD for treatment of NIF-resistant arrhythmias were included in this study, and retrospective analysis was performed. Clinical efficacy (terminative and preventive effects on arrhythmia) of intravenous AMD was evaluated. *Results*: All cases were emergent cases and had depressed left ventricular ejection fraction (30 ± 13%). Clinical arrhythmias were ventricular fibrillation (VF) in four patients, ventricular tachycardia (VT) in six patients, and atrial fibrillation (AF) in one patient. NIF was administered to all patients by intravenous injection. After administration of NIF, VT/VF/AF was terminated in seven of the 10 patients, but a preventive effect was not obtained in any of the patients (NIF-resistance). Intravenous AMD (maintenance dose: 484 ± 166 mg/day) was effective both in termination (80%) and in prevention (80%) of VT/VF events in those patients. It was also effective in termination (80%) and prevention (60%) of AF events refractory to NIF. During continuous AMD administration, no significant adverse effects or proarrhythmic effects were observed in any of the patients. Five patients died within one month, but there was no arrhythmic deaths. *Conclusions*: Intravenous AMD was effective in NIF-resistant lethal arrhythmias and was relatively safe in emergent cases with severe heart failure.

## 1. Introduction

Antiarrhythmic drugs we can use for refractory arrhythmias in emergent care settings are confined to a few types. Intravenous amiodarone hydrochloride (AMD) is a class III antiarrhythmic drug that has diverse electrophysiological actions and can block multiple channels, including the rapid component of the delayed rectifier potassium current (I_Kr_), the slowly activating delayed rectifier K^+^ currents (I_Ks_), I_Ca-L_ and I_Na_ channel and beta receptor, and has been widely used for the management of emergent patients [1-3] and is recommended in the 2005 AHA guidelines for antiarrhythmic therapy in patients with shock-refractory ventricular fibrillation (VF) [4].

Nifekalant hydrochloride (NIF) is also an intravenously administered class III antiarrythmic drug used mainly in Japan [5]. NIF selectively blocks I_Kr_ and prolongs the refractory period. NIF has been found to have a significant antiarrhythmic effect to terminate shock-refractory lethal ventricular arrhythmias [6-11]. NIF has also been reported to suppress the induction of VT and VF induced by programmed electrical stimulation [11]. NIF have several advantageous effects as a pure I_Kr_ blocker when used particularly in emergent care settings. First, NIF has no negative inotropic effect and therefore has least impact on hemodynamics. Second, NIF has rapid onset of action and clearance. Third, as with other selective I_Kr_ blockers, NIF reduces the defibrillation threshold [12]. Considering the above, it may make sense clinically to use NIF as the initial drug of choice for emergent care of lethal arrhythmias in Japan. However, NIF cannot always achieve the desired effect and we occasionally experience cases of NIF-resistant arrhythmias. Here, we report 11 severe heart failure cases with NIF-resistant lethal arrhythmia, and we examined the effects of intravenous AMD in those patients.

## 2. Materials and Methods

### 2.1. Patients

Eleven consecutive severe heart failure patients who had received intravenous AMD for treatment of NIF-resistant arrhythmias in four hospitals (Okayama University Hospital, National Hospital Organization Iwakuni Medical Center, Cardiovascular Center Sakakibara Hospital, and Okayama Redcross Hospital) between 2000 and 2008 were included in this study, and retrospective analysis was performed.

NIF was initially administered at the standard dose and the dose was increased as needed in 0.05-0.1 mg/hr increments with careful ECG monitoring so that the QTc interval would not exceed 0.55 sec^1/2^. AMD had been administered at the basis of the formally recommended dose in the US. Bolus infusion was avoided and maintenance dose was flexibly reduced depending on the patient's condition. To ensure objective assessment, physicians who were not directly involved in the treatment organized an efficacy evaluation committee and assessed each case report form.

Patients were considered to have “severe heart failure” if their condition before use of NIF or AMD was (i) New York Heart Association classification IV or (ii) left ventricular ejection fraction (LVEF)< 40%, (iii) if they needed to be managed with a cardiac support device such as a percutaneous cardiopulmonary support system (PCPS), intra-aortic balloon pumping (IABP) or a left ventricular assist device (LVAD), (iv) if they needed to be managed with intubation, (v) if they were in cardiogenic shock or (vi) if they were in cardiopulmonary arrest. The investigation conformed to the principles outlined in the Declaration of Helsinki.

### 2.2. Clinical Assessment

Twelve-lead ECG was repeatedly recorded, and heart rate, Bazzet-corrected QT interval (QTc) and blood pressure were checked at appropriate times. Patient outcome (alive or dead) was investigated 30 days after the start of drug administration.

### 2.3. Efficacy Evaluation

#### 2.3.1. Termination of Arrhythmias

NIF or AMD was assessed in terminating VT/VF or AF by the following criteria described previously [7]: (i) VT/VF or AF successfully terminated by bolus or continuous administration of NIF or AMD and (ii) VT/VF or AF not terminated by DC shock before intravenous administration of NIF or AMD but successfully terminated by additional DC shock after use of NIF or AMD (regarded as “enhancement of defibrillating effect”). Cases were judged “not evaluable” when another antiarrhythmic drug was used at the same time (or immediately after) administration of NIF or AMD.

#### 2.3.2. Prevention of Recurrence of Arrhythmias

NIF or AMD was assessed as “effective” in preventing recurrence of VT/VF or AF on the basis of the following criteria described previously: (i) complete suppression of the recurrence of sustained VT/VF or AF during maintenance infusion of the drug, (ii) recurrence during maintenance infusion of the drug but not after increase in the dose of NIF or AMD, (iii) complete suppression, maintenance infusion of the drug, of the recurrence of sustained VT/VF, which had occurred at least twice before intravenous administration of NIF or AMD. The observation time for assessment of the efficacy was set to 24 hours.

### 2.4. Safety Evaluation

We defined clinical signs and abnormal changes in laboratory test results which were thought to be related to the drugs as adverse reactions.

### 2.5. Statistics

Data are expressed as mean values ± SD. Comparisons of heart rate and QTc interval at baseline and during administration of NIF and AMD were made using one-way repeated analysis of variance followed by Bonferroni correction, and categorical data and percentage frequencies were analyzed by the chi-square test (Dr SPSS II for windows). A value of p < 0.05 was considered to be statistically significant.

## 3. Results

### 3.1. Patients' Characteristics

The patients' characteristics are summarized in Table 1. A total of 11 patients in whom arrhythmias could not be controlled by other antiarrhythmic drugs were examined. The patients included seven males and four females aged from 0.8 to 79 years (mean age: 53 ± 20 years).

Underlying heart diseases were myocarditis in two patients, acute myocardial infarction (AMI) in seven patients, dilated cardiomyopathy in one patient and non-obstructive hypertrophic cardiomyopathy in one patient. Clinical arrhythmias were VF alone in two patients, VT alone in three patients, VT/VF in one patient, VT/VF/AF in one patient, VT/AF in three patients and AF alone in one patient. Nine patients had LVEF≤ 30%, and mean LVEF was 30 ± 13%. There were nine patients who received concurrent treatment with catecholamine. Seven patients required the use of IABP and six patients required PCPS for uncontrolled VT or VF with hemodynamic collapse.

### 3.2. Intravenous Administration of NIF and AMD

A single bolus administration of NIF (0.3-0.6 mg/kg) was followed by a maintenance dose of 0.15-0.50 mg/kg/h. AMD was administered at several doses (Table 2 and Table 3). Bolus injection before continuous infusion was used in two patients and continuous infusion without bolus injection was used in nine patients. Mean maintenance doses of AMD were 484 ± 166 mg/day for VT/VF and 480 ± 110 mg/day for AF. In three patients (cases No. 5, No. 7 and No. 10), simultaneous infusion of NIF and AMD was used. In case No. 5, administration of NIF was stopped, but administration of AMD was continued, and we could therefore evaluate the efficacy of each drug independently. In the other two cases (case Nos. 7 and 10), we could not evaluate the effect of intravenous AMD independently.

### 3.3. Antiarrhythmic Efficacy for Ventricular Arrhythmias

Table 2 shows the antiarrhythmic efficacy of NIF and AMD for ventricular arrhythmias. Ten patients who suffered from VT and/or VF were evaluated. NIF was administered to all patients at a mean dose of 0.39 ± 0.14 mg/kg as a bolus and at 0.34 ± 0.12 mg/kg/h as continuous infusion. After administration of NIF, VT/VF was terminated in seven of the 10 patients, but not preventive effect was obtained in any of the patients (NIF-resistance). During continuous NIF administration, TdP associated with excessive QT prolongation (0.670 sec^1/2^) was observed in one case (case No. 3). AMD alone terminated VT/VF in 8 (80%) of the 10 patients, and prevention of VT/VF was also achieved in eight (80%) of the 10 patients. In the other two patients (cases No. 7 and No. 10), both termination and prevention were achieved by simultaneous administration of NIF and AMD. Five of the 10 patients in our study died within one month, but there were no arrhythmic deaths (Table 1).

### 3.4. Antiarrhythmic Efficacy for Atrial Arrhythmias

Table 3 shows the antiarrhythmic efficacy of NIF and AMD for AF. AF was paroxysmal in all cases. Four patients who suffered from AF also had VT or VF, but none of the VT or VF episodes were promoted by AF. NIF was used in all patients at a mean dose of 0.36 ± 0.13 mg/kg as a bolus and at 0.34 ± 0.09 mg/kg/h as continuous infusion. Neither a terminative nor preventive effect was observed after administration of NIF in any of the patients (NIF-resistance). Intravenous AMD alone (continuous infusion at a mean dose of 480 ± 110 mg/day) resulted in termination of AF in four (80%) of the five patients and in prevention of AF in three (60%) of the five patients. In one patient (case No. 7), termination of AF was achieved by simultaneous administration of NIF and AMD.

### 3.5. Effects on Heart Rate and ECG Parameters

**Figure 1:**
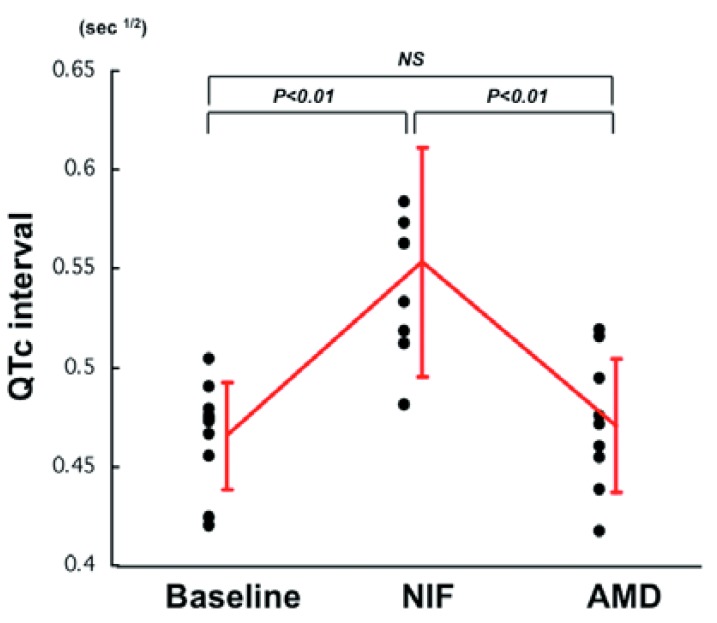
Effects of administration of nifekalant hydrochloride (NIF) and intravenous amiodarone (AMD) on QTc interval.

Table 4 shows heart rate (HR) and QTc interval in all patients during administration of NIF and AMD. HR did not change during administration of NIF but significantly decreased after administration of AMD (NIF: 100 ± 35 *vs.* AMD: 69 ± 15/min, P < 0.05). Significant QTc prolongation was observed during NIF administration (*vs.* baseline, p < 0.01), but there was no difference in QTc interval during AMD administration (*vs.* baseline, p = NS) (Table 4 and Figure 1).

In three cases for which simultaneous administration of NIF and AMD was performed, AMD further prolonged the QTc interval that had already been prolonged by NIF (baseline: 0.466 ± 0.027, NIF: 0.554 ± 0.058, NIF + AMD: 0.577 ± 0.032 sec^1/2^).

### 3.6. Adverse Effects

During continuous NIF administration, TdP associated with excessive QT prolongation was observed in one case (case No. 3). During continuous AMD administration, significant adverse effects, including lung fibrosis and liver dysfunction, proarrhythmic effects and worsening of cardiac function were not observed in any of the patients in this study.

## 4. Discussion

The optimal use of antiarrhythmic drugs for refractory arrhythmia with severe heart failure has not been established. While both AMD and NIF have been reported to be efficacious for refractory arrhythmias, there has been no study on the effect of AMD on NIF-resistant arrhythmia in patients with severe heart failure. In this study, we found that intravenous AMD, a multi-channel (I_Kr_, I_Ks_, I_Na_ and I_Ca-L_) and beta receptor blocker [3], was effective for NIF (a selective I_Kr_ blocker)-resistant lethal arrhythmias in emergent cases with severe heart failure.

QTc interval was prolonged by NIF but not by AMD alone. Therefore, QT prolongation was not effective for inhibition of arrhythmias in these cases. Interestingly, we found that the QTc interval was prolonged by simultaneous use of NIF and AMD. This could be explained by the reverse use-dependent property of NIF, which means that the I_Kr_ blocking effect of NIF was augmented by decrement of HR caused by AMD. Thus, caution is needed in simultaneous use of NIF and AMD.

The present study showed high mortality, but during continuous AMD administration, significant adverse effects, including lung fibrosis and liver dysfunction, proarrhythmic effects and worsening of cardiac function were not observed in any of the patients in this study. However, long term treatment with amiodarone has no favorable effect on survival [13]. Careful use of amiodarone is needed in patients with severe heart failure.

AMD was administered when NIF was not effective for termination or prevention of VT/VF in this study. On the other hand, NIF might be effective in different cases that do not respond to intravenous AMD. Further studies are needed to clarify this point.

## 5. Limitations

Since the present study was a retrospective study, it has several limitations. First, it involves a small number of patients from four hospitals over eight years with substantial inhomogeneity in the patients' background (ages from 1 year to 79 years), presenting arrhythmias (VT, VF or AF) and method of treatment (simultaneous or subsequent, with or without bolus infusion and) at varying doses of drugs in this study. This might have affected the efficacy of drugs. Second, since all of the 11 patients in this study were treated with other antiarrhythmic drugs before treatment with NIF and AMD, an additive effect cannot be ruled out. Therefore it is unclear whether the efficacy on arrhythmia and the change in ECG parameters in this study were due to either drug, combination of both drugs or other factors. To elucidate the interaction and joint effectiveness of a combination of these drugs, a prospective trial with treatment groups and well defined dosing regimens would be more suitable, albeit difficult to undertake under emergency conditions. Even for retrospective study, larger numbers and more stringent inclusion criteria would be required. Further studies are needed to clarify this point.

## 6. Conclusions

Intravenous AMD was effective in NIF-resistant lethal arrhythmias and was relatively safe in emergent cases with severe heart failure.

## Figures and Tables

QTc interval was increased after NIF administration but returned to baseline level after AMD administration.

**Table 1:** Patients' characteristics.

**Case No.**	**Age (y)**	**Gender**	**Heart disease**	**LVEF (%)**	**Arrhthymia**	**CA**	**IABP**	**PCPS**	**Outcome**
1	0.8	Female	myocarditis	25	VT	−	−	−	alive
2	50	Female	myocarditis	27	VT	−	−	−	alive
3	48	male	DCM	25	VT/VF, AF	−	−	−	dead (thrombosis)
4	63	male	HCM	65	VF	−	−	−	alive
5	65	male	AMI	30	VF	−	−	−	alive
6	62	male	AMI	30	VT,AF	−	−	−	dead (infection)
7	65	male	AMI	25	VT, AF	−	−	−	dead (MOF)
8	57	male	AMI	20	VT	−	−	−	dead (MOF)
9	45	male	AMI	18	VT, AF	−	−	−	dead (MOF)
10	50	Female	AMI	30	VT/VF	−	−	−	alive
11	79	Female	AMI	37	AF	−	−	−	alive

LVEF, left ventricualr ejection fraction; CA, catecholamine; IABP, intraaortic baloon pumping; PCPS, percutaneous cardiopulmonary support; DCM, dilated cardiomyopathy; HCM, hypertrophic cardiomyopathy; AMI, acute myocardial infarction; VT, ventricular tachycaridia; VF, ventricular fibrillation; AF, atrial fibrillation; MOF, multiple organ failure.

**Table 2:** Effects of class-III drugs on ventricular arrhythmias.

**Case No.**	**Dose of NIF**	**termination**	**prevention**	**Dose of AMD**	**termination**	**prevention**
1	0.3 mg/kg bolus + 0.5 mg/kg/h	+	−	140 mg/day	+	+
2	0.3 mg/kg bolus + 0.4 mg/kg/h	−	NE	300 mg/day	+	+
3	0.6 mg/kg bolus + 0.4 mg/kg/h	+	−	600 mg/day	+	+
4	0.3 mg/kg bolus + 0.4 mg/kg/h	−	NE	125 mg bolus + 300-600 mg/day	+	+
5	0.3 mg/kg bolus + 0.4 mg/kg/h	+	−	600 mg/day	+	+
6	0.3 mg/kg bolus + 0.4 mg/kg/h	+	−	600 mg/day	+	+
7	0.3 mg/kg bolus + 0.3 mg/kg/h	−	NE	400 mg/day	NE*	NE*
8	0.6 mg/kg bolus + 0.2 mg/kg/h	+	−	150 mg bolus + 600 mg/day	+	+
9	0.3 mg/kg bolus + 0.2 mg/kg/h	+	−	400 mg/day	+	+
10	0.6 mg/kg bolus + 0.15 mg/kg/hr	+	−	600 mg/day	NE*	NE*

mean	0.39 ± 0.14 mg/kg bolus + 0.34 ± 0.12 mg/kg/h			484 ± 166 mg/day		

AMD, amiodarone; NIF, nifekalant hydrochloride; NE, not evaluated;

* effective under the condition of simultaneous administration of AMD and NIF.

**Table 3:** Effects of class III drugs on atrial arrhythmias.

**Case No.**	**Dose of NIF**	**termination**	**prevention**	**Dose of AMD**	**termination**	**prevention**
3	0.6 mg/kg bolus + 0.4 mg/kg/h	−	−	600 mg/day	+	+
6	0.3 mg/kg bolus + 0.4 mg/kg/h	−	−	600 mg/day	+	−
7	0.3 mg/kg bolus + 0.3 mg/kg/h	−	−	400 mg/day	NE*	−
9	0.3 mg/kg bolus + 0.2 mg/kg/h	−	−	400 mg/day	+	+
11	0.3 mg/kg bolus + 0.4 mg/kg/h	−	−	400 mg/day	+	+

mean	0.36 ± 0.13 mg/kg bolus + 0.34 ± 0.09 mg/kg/h			480 ± 110 mg/day		

AF, atrial fibrillation; AMD, intravenous amiodarone; NIF, nifekalant hydrochloride; NE, not evaluated;

* effective under the condition of simultaneous administration of NIF and AMD.

**Table 4:** Electrocardiogaphical parameters.

**Case No.**	**Baseline**		**NIF**		**NIF+ AMD**†		**AMD**	

	HR (/min)	QTc (sec^1/2^)	HR (/min)	QTc (sec^1/2^)	HR (/min)	QTc (sec^1/2^)	HR (/min)	QTc (sec^1/2^)
1	181	0.455	176	0.583			75	0.515
2	95	0.479	NE	NE			100	0.438
3	86ˆ	0.490	86	0.670			52	0.519
4	VF	VF	VF	VF			58	0.417
5	102	0.472	76	0.518	78	0.547	63	0.454
6	50	0.475	81	0.512			80	0.471
7	94	0.466	NE	NE	90	0.574	NE	NE
8	96	0.504	81	0.562			74	0.494
9	84	0.474	120ˆ	0.573			59	0.475
10	97	0.420	107	0.481	60	0.610	NE	NE
11	62	0.424	75	0.533			60	0.460
mean	95 ± 35	0.466 ± 0.027	100 ± 35	0.554 ± 0.058*	76 ± 15	0.577 ± 0.032	69 ± 15	0.471 ± 0.034**

HR, heart rate; QTc, corrected QT interval; AMD, amiodarone; NIF, nifekalant; NE, not evaluated; VF, ventricular fibrillation;

† simultaneous administration of NIF and AMD;

ˆ status of AF;

* P < 0.01 *vs.* baseline,

** P < 0.05 *vs.* NIF.

## References

[1] Dorian P., Cass D., Schwartz B., Cooper R., Gelaznikas R., Barr A. (2002). Amiodarone as compared with lidocaine for shock-resistant ventricular fibrillation. N. Engl. J. Med..

[2] Kudenchuk P.J., Cobb L.A., Copass M.K., Cummins R.O., Doherty A.M., Fahrenbruch C.E., Hallstrom A.P., Murray W.A., Olsufka M., Walsh T. (1999). Amiodarone for resuscitation after out-of-hospital cardiac arrest due to ventricular fibrillation. N. Engl. J. Med..

[3] Kodama I., Kamiya K., Toyama J. (1997). Cellular electropharmacology of amiodarone. Cardiovasc. Res..

[4] (2005). 2005 American Heart Association Guidelines for Cardiopulmonary Resuscitation and Emergency Cardiovascular Care. Circulation.

[5] Nakaya H., Tohse N., Takeda Y., Kanno M. (1993). Effects of MS-551, a new class III antiarrhythmic drug, on action potential and membrane currents in rabbit ventricular myocytes. Br. J. Pharmacol..

[6] Yoshioka K., Amino M., Morita S., Nakagawa Y., Usui K., Sugimoto A., Matsuzaki A., Deguchi Y., Yamamoto I., Inokuchi S. (2006). Can nifekalant hydrochloride be used as a first-line drug for cardiopulmonary arrest (CPA)? : Comparative study of out-of-hospital CPA with acidosis and in-hospital CPA without acidosis. Circ. J..

[7] Katoh T., Mitamura H., Matsuda N., Takano T., Ogawa S., Kasanuki H. (2005). Emergency treatment with nifekalant, a novel class III anti-arrhythmic agent, for life-threatening refractory ventricular tachyarrhythmias: post-marketing special investigation. Circ. J..

[8] Tahara Y., Kimura K., Kosuge M., Ebina T., Sumita S., Hibi K., Toyama H., Kosuge T., Moriwaki Y., Suzuki N. (2006). Comparison of nifekalant and lidocaine for the treatment of shock-refractory ventricular fibrillation. Circ. J..

[9] Washizuka T., Chinushi M., Watanabe H., Hosaka Y., Komura S., Sugiura H., Hirono T., Furushima H., Tanabe Y., Aizawa Y. (2005). Nifekalant hydrochloride suppresses severe electrical storm in patients with malignant ventricular tachyarrhythmias. Circ. J..

[10] Yusu S., Ikeda T., Mera H., Miyakoshi M., Miwa Y., Abe A., Tsukada T., Ishiguro H., Shimizu H., Yoshino H. (2009). Effects of intravenous nifekalant as a lifesaving drug for severe ventricular tachyarrhythmias complicating acute coronary syndrome. Circ. J..

[11] Kondoh K., Hashimoto H., Nishiyama H., Umemura K., Ozaki T., Uematsu T., Nakashima M. (1994). Effects of MS-551, a new class III antiarrhythmic drug, on programmed stimulation-induced ventricular arrhythmias, electrophysiology, and hemodynamics in a canine myocardial infarction model. J. Cardiovasc. Pharmacol..

[12] Murakawa Y., Yamashita T., Kanese Y., Omata M. (1997). Can a class III antiarrhythmic drug improve electrical defibrillation efficacy during ventricular fibrillation?. J. Am. Coll. Cardiol..

[13] Bardy G.H., Lee K.L., Mark D.B., Poole J.E., Packer D.L., Boineau R., Domanski M., Troutman C., Anderson J., Johnson G. (2005). Amiodarone or an implantable cardioverter-defibrillator for congestive heart failure. N. Engl. J. Med..

